# Single piece craniotomy in the management of a patient with large vertex epidural haematoma and deteriorating brain function in a limited resources community. Case report from Southwestern Uganda

**DOI:** 10.1016/j.ijscr.2025.111146

**Published:** 2025-03-14

**Authors:** Robert Mugarura, Irene K. Nakitto, Nek A. Jonathan, Naphtali Kamusiime, Mary Katushabe

**Affiliations:** a Department of Surgery, Kabale University School of Medicine, Uganda; b Department of Public Health, Kabale University, Uganda; c Bethel SurgiCO Clinic, Kabale, Uganda; d Kisiizi COU hospital, Rukungiri, Uganda; e Public Health Nurse specialist, Muhumure Healthcare Foundation, Uganda

**Keywords:** Vertex epidural haematoma, Single piece craniotomy, Massive haemorrhage, Rural communities, Southwestern Uganda, Case report

## Abstract

**Introduction and importance:**

Vertex epidural haematoma is a rare form of epidural haemorrhage and a complication of head trauma that is both a diagnostic and therapeutic challenge for the trauma surgeon. It's usually as result of laceration in the superior sagittal sinus. Whereas most patients with small vertex epidural hematomas improve with conservative treatment, large or rapidly expanding lesions are fatal and require emergency craniotomy.

**Case presentation:**

Ours was a 28-year-old male patient who arrived at our emergency department in rural Southwestern Uganda with Glasgow coma scale of 12/15 following a head on collision of two Boda Boda motorcycles. CT scan imaging revealed a large acute Vertex Epidural haematoma and a linear skull fracture of the parietal bones crossing the midline. The haematoma was evacuated through a single piece craniotomy and hemostasia achieved by surgicel. Patient was transfused with two units of whole blood. Patients Glasgow coma scale improved to 15/15 after 24 h postoperatively.

**Clinical discussion:**

Vertex epidural hematomas are both diagnostic and therapeutic challenges. Small haematomas do not require surgical intervention. Large and expanding haematomas are fatal and require urgent surgical evacuation by neurosurgeons. In rural centres in lower- and middle-income countries like our own, surgery is often more challenging. This is mainly due to nonavailability of neurosurgeons and lack of medical supplies. With careful planning, available midlevel medical doctors can safely evacuate a large vertex epidural hematoma through a single piece craniotomy.

**Conclusion:**

When performed timely, emergency surgical decompression results into rapid improvement of brain function in patients with large vertex epidural hematoma and deteriorating brain function as observed in our patient. Massive haemorrhage from superior sagittal sinus and air embolism are important intraoperative complications that should be kept in mind. Careful pre operative planning is of paramount importance especially in centres without neurosurgeons and essential neurosurgery supplies.

## Highlights

• Vertex epidural hematoma is rare and is often missed during clinical assessment of trauma patients
• Large and rapidly expanding vertex epidural haematoma are fatal and require emergency surgical evacuation.
• In rural communities in southwestern Uganda with limited access to surgical care and no neurosurgeons, vertex epidural hematomas are treated by non specialist medical practitioners
• Single piece craniotomy is simple and quick to perform using basic neurosurgical equipment by a non-neurosurgical specialist.
• Massive haemorrhage from superior sagittal sinus and air embolism are important intraoperative complications that should be kept in mind.
• Careful preoperative preparation reduces morbidity and Mortality from intraoperative complications.

## Abbreviations

CT: Computer Tomography
GCS: Glasgow Coma Scale
ICP: Intracranial Pressure
SSS: Superior Sagittal Sinus
VEDH: Vertex epidural hematoma

## 1 Introduction

Vertex epidural hematoma (VEDH) is a rare complication of head trauma accounting for 1.3–8.2 % of all traumatic intracranial hematomas [1].

The most common source of bleeding is laceration in superior sagittal sinus (SSS). Other documented sources of bleeding include; fracture line, dural stripping from the inner table of the skull and the diseased vascular skull bone in Paget's disease. Spontaneous bleeding has also been reported [2].

VEDH possess both diagnostic and therapeutic challenges for the neurosurgeon because of its pathogenesis and anatomical location. The vertex has been referred as a radiological “blind spot” [3]. As a result, VEDH may be missed on CT scan imaging. When missed or misdiagnosed, large VEHDs have a mortality of up to 50 % [4].

There are no reported cases of VEDH in rural communities in low middle income countries in Africa. This may be because VEDHs are often missed or misdiagnosed in limited resource health facilities like ours.

Large or rapidly evolving hematomas are fatal and require urgent surgical evacuation. Surgical evacuation carries potentially fatal complications. These include massive haemorrhage from SSS laceration and air embolism. These complications may require specialist neurosurgical skills to manage. In rural communities without neurosurgeons like our own, surgical evacuation can be very challenging.

Careful preoperative preparation, patient optimization and the choice of surgical approach is key to successfully evacuate VEDHs while at the same time minimizing intraoperative complications.

This case report of a patient with VEDH treated by emergency surgical evacuation of the haematoma in our centre has been reported in line with the SCARE criteria [5].

## 2 Case presentation

**Fig. 1:**
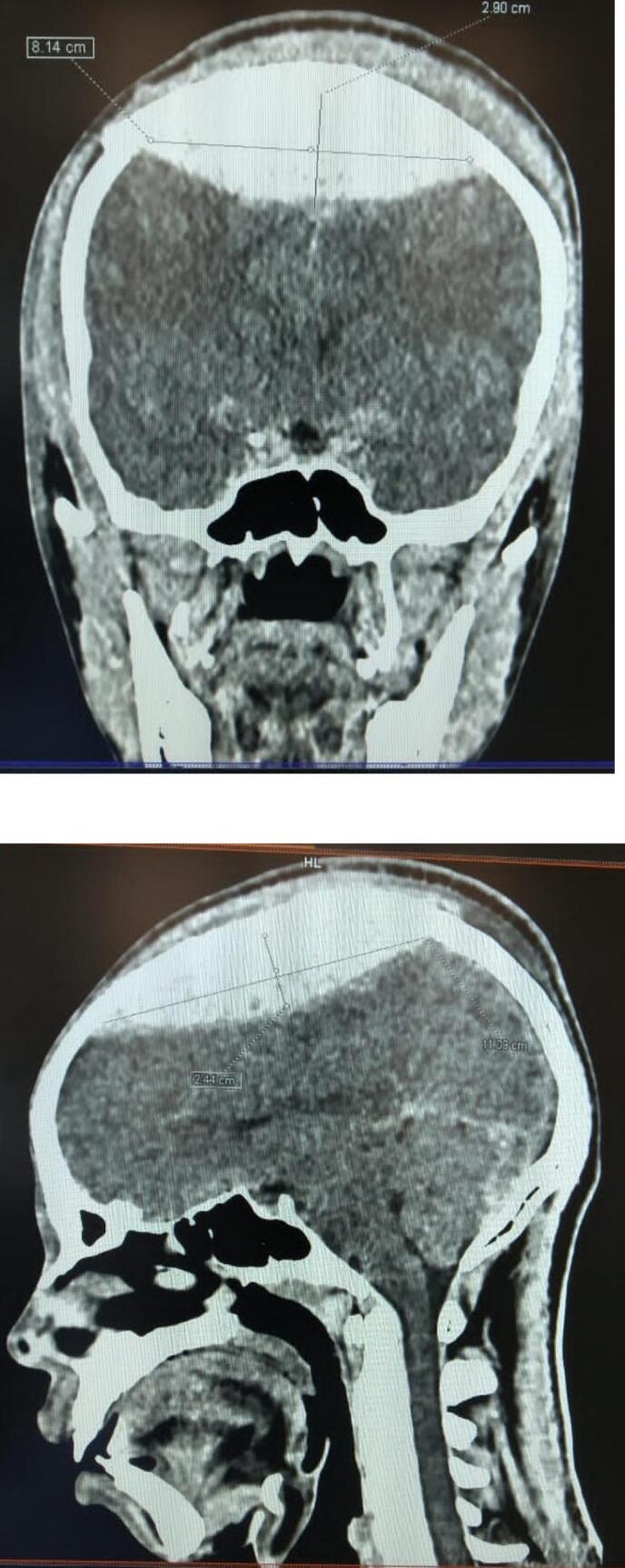
CT scan images showing large acute vertex epidural haematoma crossing the midline.

**Fig. 2:**
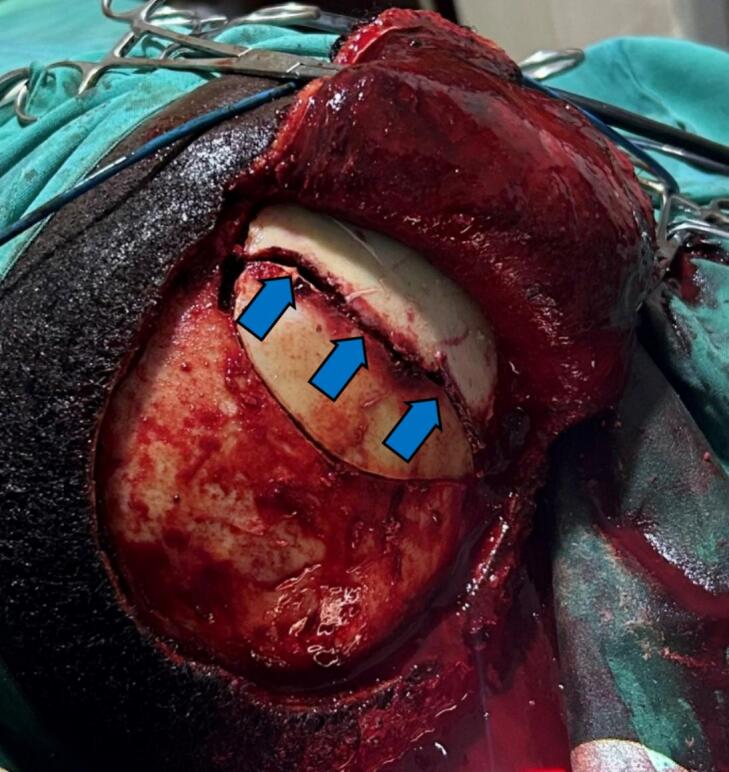
Linear skull fracture on craniotomy (with blue arrows). (For interpretation of the references to colour in this figure legend, the reader is referred to the web version of this article.)

We present a 28-year-old male patient who arrived at our emergency department in rural Southwestern Uganda with reduced level of consciousness and Glasgow coma scale of 12/15. This followed head on collision of two Boda Boda motorcycles 36 h prior to presenting to our emergency department one of which he was the rider. Both motorcycles had no passengers. CT scan Imaging revealed a large acute VEDH (8 cm wide, 11 cm long, and 2.9 cm deep) and a linear skull fracture of the parietal bones crossing the midline (Fig. 1, Fig. 2). There was no midline shift but significant brain edema.

Prompt surgical evacuation was deemed necessary owing to the size of the haematoma and the reduced GCS. Four units of whole blood were booked and surgicel was bought from the nearest big medical store which is 140 km away.

**Fig. 3:**
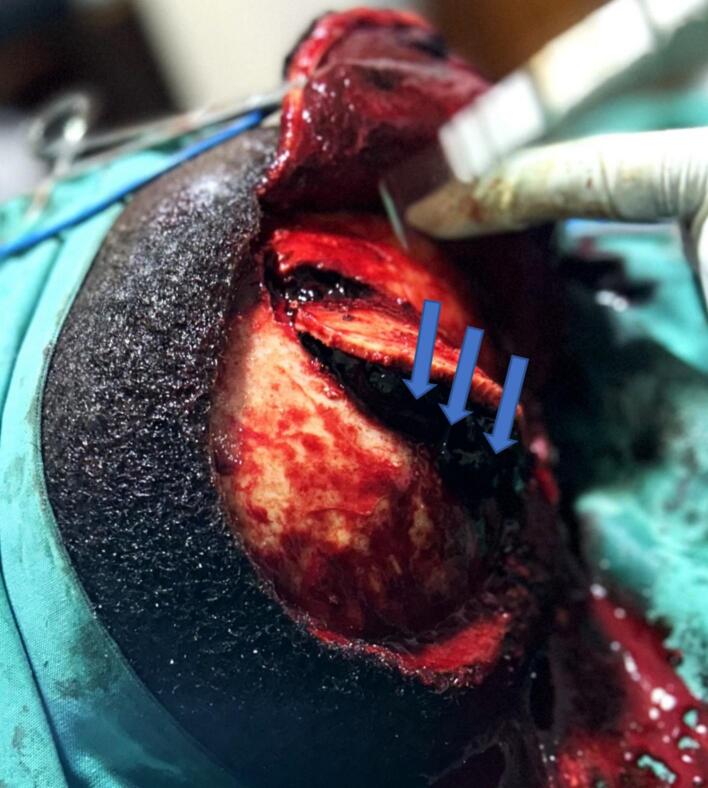
Large Vertex Epidural haematoma under pressure lifting the bone flap after craniotomy (Blue arrows). (For interpretation of the references to colour in this figure legend, the reader is referred to the web version of this article.)

We performed a single piece craniotomy crossing the midline over the haematoma. We found a large VEDH under pressure (Fig. 3).

We gently evacuated the hematoma under normal saline irrigation taking care to not to remove all the clot over the SSS. Even with careful attention, significant haemorrhage was encountered intraoperatively from the SSS requiring blood transfusion. Haemostasis was achieved with Surgicel (Ethicon) and patient transfused with two units of whole blood. No other complications were encountered. The patient GCS improved to 15/15 within 24 h postoperative period.

## 3 Discussion

VEDHs are mainly of venous origin. Venous-originated epidural hematomas grow slowly and often under go early coagulation. As a result, most patients with VEDHs can be managed conservatively with no surgery compared to epidural hematomas originating from arterial bleeding [6]. Decision to manage VEDHs nonoperatively must be individualized and careful patient monitoring should be undertaken.

In the largest case series of 29 patients reported, 4 patients (13.8 %) required emergency craniotomy [7]. Whereas majority of patients with VEDH may improve on conservative treatment, surgery when required should be performed urgently and without any delay.

Indications for emergency craniotomy in a patient with VEDH are; deteriorating level of consciousness, features of severely increased intracranial pressure (ICP), focal neurological deficit and hematoma more than 30 ml in volume [8]. Our patient had deteriorating level of consciousness and thus the decision to convert from conservative to surgical treatment.

Various surgical approaches for evacuation of VEDH have been described. These include; primary burr holes, one-piece craniotomy across the midline and biparietal parasagittal craniotomy with a strip of central bone.

Whereas primary burr holes are easy to perform, they are limited in achieving hematoma evacuation, haemostasis and are more susceptible for re-accumulation of blood, and might require additional surgery [9].

Biparietal parasagittal craniotomy with a central bone strip carries the advantage of avoiding the use of the craniotome over the potentially torn sagittal sinus and preserves a bar of bone over the sinus to allow effective dural hitch and tamponade whilst still allowing full evacuation of the hematoma [10]. Biparietal parasagittal craniotomy however requires specialist surgical skills, longer operating time, and specialized neurosurgical equipment.

Single piece craniotomy carries the advantages of short operative time, direct exposure of the whole hematoma taking into consideration that the sinus is already pushed away downward by the hematoma and itself causing tamponade effect on the sinus so the risk of facing early sinus bleeding is less [11]. Single piece craniotomy can also easily be performed using basic neural instruments like manual hand-held Hudson drill and Gigli saw. We decided to use the single piece craniotomy which gives quick access to the hematoma and source of bleeding using basic surgical instruments.

In centres with limited access to surgical care [12] and no neurosurgeons like our own, patients with intracranial bleeding are often treated by nonspecialist medical practitioners.

As encountered in our case, control of intraoperative haemorrhage from the SSS which is often the source of VEDH can be challenging to the general surgeon or nonspecialist medical doctor. Adequate preoperative preparation is of paramount importance to minimise adverse outcomes that may arise from intraoperative complications.

Carefully planned surgical evacuation of VEDH and haemostasis results into rapid recovery of brain function as was observed for our patient.

## 4 Conclusion

Emergency single piece craniotomy can improve treatment outcomes in patients with large VEDH and deteriorating brain function. The most common intraoperative complication is massive haemorrhage from the superior sagittal sinus laceration. Careful preoperative planning is necessary to minimise and manage intraoperative complications.

## CRediT authorship contribution statement

Conception and design – Robert Mugarura, Nek Arthur Jonathan

Drafting the article and revising it critically for important intellectual content - Robert Mugarura, Nek Arthur Jonathan, Nakitto Irene Kisakye, Naphthali Kamusiime, Mary Katushabe

Final approval of the version published - Robert Mugarura, Nek Arthur Jonathan, Nakitto Irene Kisakye, Naphthali Kamusiime, Mary Katushabe

Agreement to be accountable for the article and to ensure that all questions regarding the accuracy or integrity of the article are investigated and resolved - Robert Mugarura, Nek Arthur Jonathan

## Consent for publication

Written informed consent was obtained from the patient for publication and any accompanying images. A copy of the written consent is available for review by the Editor-in-Chief of this journal on request.

## Ethical approval

Ethical approval was waived by Kabale university Research Ethics Committee (KAB-REC). Case reports on a single patient encountered during clinical care do not constitute research at our institution and therefore are exempted from ethics approval. However informed written consent to use information and images for educational purposes and publication must be obtained from the patient.

Written consent was obtained from the patient.

## Guarantor

Robert Mugarura.

## Research registration number

Not applicable.

## Funding

This case report received no specific grant from any funding agency in the public, commercial, or not-for-profit sectors.

## Declaration of competing interest

The authors declare that there is no conflict of interest.

## Data Availability

Supporting data is available upon request.
